# An algorithmic characterization and spectral analysis of canonical splitting signed graph ξ(Σ)

**DOI:** 10.1016/j.mex.2023.102517

**Published:** 2023-12-15

**Authors:** Deepa Sinha, Sandeep Kumar

**Affiliations:** Department of Mathematics, Faculty of Mathematics and Computer Science, South Asian University, New Delhi 110068, India

**Keywords:** Signed graph, Splitting signed graph, Spectrum, Energy, Graph algorithm

## Abstract

An ordered pair $\Sigma =(\Sigma^{u},\sigma )$ is called the *signed graph*, where $\Sigma^{u}\;=\;\left(V,\;E\right)$ is an underlying *graph* and σ is a signed mapping, called *signature*, from E to the sign set {+,−}. A *marking* of Σ is a function μ:V(Σ)→{+,−}. The *canonical marking* of a signed graph Σ, denoted $\mu_{\sigma }$, is given as

$\mu_{\sigma }\left(v\right)\;=\;\Pi_{vu\in E(\Sigma )}\sigma \left(vu\right)$.

The *canonical splitting signed graph*ξ(Σ) of a signed graph Σ is defined as a signed graph ξ(Σ)=(V(ξ),E(ξ)) , with $V\left(\xi \right)\;=\;V\left(\Sigma \right)\cup V^{\prime }$, where $V^{\prime }$  is copy of a vertex set in V(Σ) s.t. for each vertex u∈V(Σ), take a new vertex $u^{\prime }$ and E(ξ) is defined as, join $u^{\prime }$ to all the vertices of Σ adjacent to u by negative edge if $\mu_{\sigma }\left(u\right)\;=\;\mu_{\sigma }\left(v\right)\;=\;-$, where v∈N(u) and by positive edge otherwise. The objective of this paper is to propose an algorithm for the generation of a canonical splitting signed graph, a splitting root signed graph from a given signed graph, provided it exists and to give the characterization of balanced canonical splitting signed graph. Additionally, we conduct a spectral analysis of the resulting graph. Spectral analysis is performed on the adjacency and Laplacian matrices of the canonical splitting signed graph to study its eigenvalues and eigenvectors. A relationship between the energy of the original signed graph Σ and the energy of the canonical splitting signed graph ξ(Σ) is established.

• Algorithm to generate canonical splitting signed graph ξ(Σ).
• Spectral Analysis is performed for both adjacency and Laplacian matrices of canonical splitting signed graph ξ(Σ).

## Graphical abstract

**Figure:**
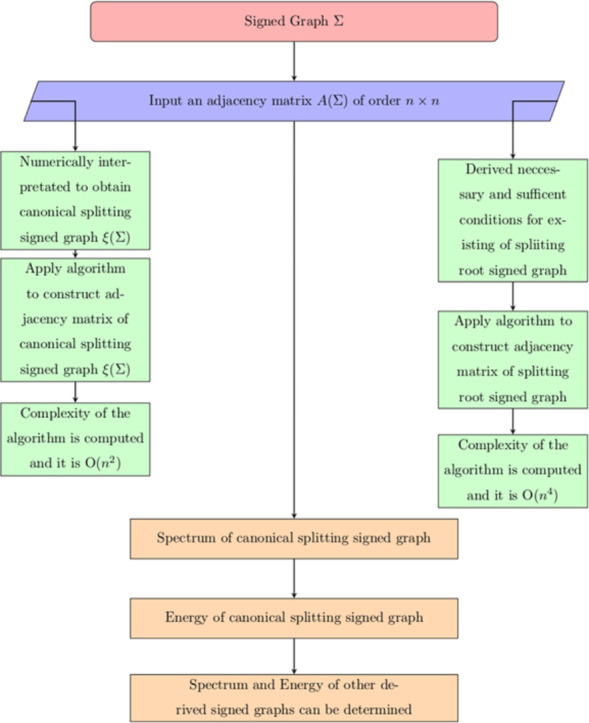

Specifications table

**Table:** 

Subject area:	Mathematics
More specific subject area:	Graph Theory
Name of your method:	Graph algorithm
Name and reference of original method:	N/A
Resource availability:	Provided in the Article

**Method details**

## Introduction

The initial notation and terminology used in this paper have been sourced from Harary [1], Zaslavsky [2] and West [3]. The graphs examined in this paper are finite and simple. A signed graph, $\Sigma \;=\;(\Sigma^{u},\;\sigma )$, is composed of an underlying graph, $\Sigma^{u}\;=\;\left(V,\;E\right)$, where |V|=n & |E|=m, and a signature, σ:E→{+,−}, which labels each edge of Σ as either ‘+’ or ‘−’. In this paper, edges labeled with ‘+’ are considered positive and are depicted using solid lines, while edges labeled with ‘−’ are considered negative and are depicted using dashed lines. If all edges in Σ are signed ‘+’ or ‘−’, the signed graph is referred to as homogeneous, otherwise, it is heterogeneous. Graphs can be thought of as homogeneous signed graphs with each edge being labeled as ‘+’. A cycle in a signed graph Σ is considered positive if it includes an even number of negative edges. If every cycle in Σ is positive, then Σ is defined as a balanced signed graph.

An ordered pair (Σ,μ) is known as a marked signed graph where $\Sigma =(\Sigma^{u},\sigma )$ is a signed graph, and $\mu \;:V\;\left(\Sigma^{u}\right)\to \;\left\{+,-\right\}$, is a function defined on the vertex set $V(\Sigma^{u})$ of $\Sigma^{u}$. The function μ assigns each vertex of $\Sigma^{u}$ to either the positive or negative sign from the set {+,−}, and is called the *marking* of Σ.

The adjacency matrix of Σ, whose vertices are $v_{1},\;v_{2},\;...,\;v_{n}$ is the n×n matrix $A\left(\Sigma \right)=\;\left[a_{i,j}\right]$ where

(1) $$a_{i,j}=\left\{ \begin{array}{cc} 0 & if\;v_{i}\;and\;v_{j}\;are\;not\;adjcent\\ 1 & if\;\sigma \left(v_{i},v_{j}\right)\;is\;postive\\ -1 & if\;\sigma \left(v_{i},v_{j}\right)\;is\;negative \end{array}\right.$$

The *spectrum* of a matrix is a list of its eigenvalues along with their multiplicities. Since, A(Σ) is a symmetric matrix with real entries so all its eigenvalues are real. Let $\lambda_{1}\left(\Sigma \right)\;>\;\lambda_{2}\left(\Sigma \right)\;>\;...\;>\;\lambda_{k}\left(\Sigma \right)$ are distinct eigenvalues of A(Σ) along with their multiplicities $m_{1},\;m_{2},\;...,\;m_{k},\;1\;\leq \;k\;\leq \;n$, then the list of eigenvalues of adjacency matrix is called *adjacency spectrum* of the signed graph Σ and usually denoted as:

$$\text{Sp}\left(\Sigma \right)=\left(\begin{array}{c} \;\lambda_{1}\;\lambda_{2}\;...\;\lambda_{k}\;\\ m_{1}\;m_{2}\;...\;m_{k} \end{array}\right)$$

Let $D\left(\Sigma \right)\;=\;\left[d_{i,\;j}\right]$ be a diagonal matrix of order n such that the entry (i,j) is deg (*u_i_*) if i=j and 0 otherwise, where deg (*u_i_*) denotes the degree of vertex *u_i_*. D(Σ) is called *degree matrix* of the signed graph Σ. The *Laplacian matrix* $L\left(\Sigma \right)\;=\;\left[l_{i,\;j}\right]$ of a signed graph Σ is a square matrix of order n such that $l_{i,\;j}\;=\;d_{i,\;j}-a_{i,\;j}$ for 0≤i,j≤n. The eigenvalues of the Laplacian matrix is called *Laplacian spectrum* and is denoted by SLp(Σ). Two signed graphs are said to be co-spectral if they have same spectrum. The largest eigenvalue $\lambda_{1}\left(\Sigma \right)$ is called the index of Σ, whereas the largest absolute eigenvalue is called spectral radius ρ(Σ), i.e.

(2) $$\rho \;=\;\text{max}\left\{\lambda_{1}\left(\Sigma \right),\;-\lambda_{k}\left(\Sigma \right)\right\}.$$

The study of graph spectrum is of significant importance in the field of graph theory, and spectral graph-theoretic techniques have been applied in a range of fields including quantum physics, chemistry, computer science, and more.

In recent years, researchers have explored the spectral properties of graphs constructed through graph operations such as disjoint union, Cartesian product, Kronecker product, strong product, lexicographic product, corona, edge corona, and neighbourhood corona. A comprehensive overview of results on the spectra of these graphs can be found in the literature [[4], [5], [6], [7], [8], [9], [10], [11], [12]] and one can find the properties of derived signed graphs in [[13], [14], [15], [16], [17], [18]]. In [19] authors presented the idea of the splitting graph $\Gamma (\Sigma^{u})$ for a given graph $\Sigma^{u}$. The process of creating the splitting graph $\Gamma (\Sigma^{u})$ involves taking a new vertex $v^{\prime }$ for each vertex v in graph $\Sigma^{u}$. The new vertex $v^{\prime }$ is then connected to all vertices in $\Sigma^{u}$ that are adjacent to v. The resulting graph is referred to as the splitting graph $\Gamma (\Sigma^{u})\;$of graph $\Sigma^{u}$.

Recently, a variation of this concept has been applied in the analysis of online social networks (OSNs), where $v^{\prime }$ is also connected to v. This variation of the concept is referred to as the “clone” of v. For the purpose of convenience, the term “clone” is adopted for $v^{\prime }$ in the splitting graph $\Gamma (\Sigma^{u})$ as well. By leveraging the signed properties of splitting signed graphs, it becomes possible to develop an alternative approach to encryption and decryption, which can be employed to enhance network security.

Gutman [20] introduced the concept of energy of a graph $\Sigma^{u}$ in 1978 as the sum of the absolute values of its eigenvalues, denoted by $E(\Sigma^{u})$, i.e.,

$$E\left(\Sigma^{u}\right)=\sum_{i=1}^{n}\left|\lambda_{i}\right|$$

**Fig. 1:**
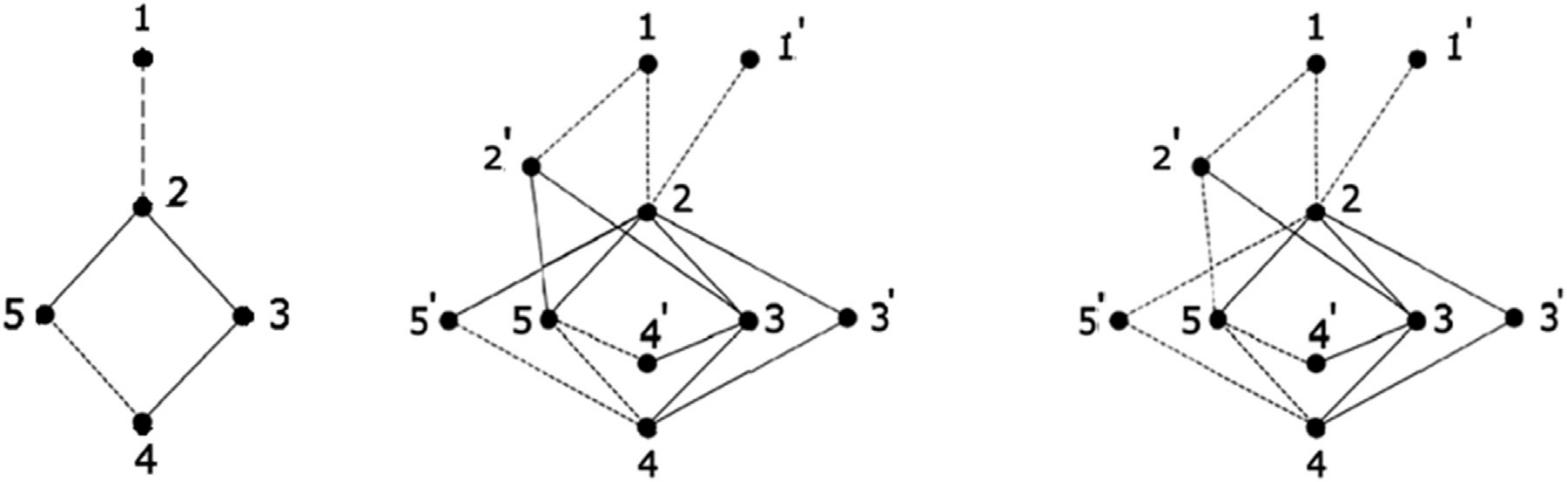
Signed graph Σ and its splitting signed graph Γ(Σ)(middle) and C−splittingsignedgraphξ(Σ).

Later, in 2004, Bapat et al. [21] proved that the energy of a graph can only be an even integer if it is a rational number. Pirzada et al. [22], on the other hand, demonstrated that the energy of a given graph can never be the square root of an odd integer. Graph energy is briefly discussed in [8], while in [23] authors established a relationship between the energy of a graph and its splitting graph. The concept of graph energy has been widely studied in graph theory and has significant applications in various fields. The study of graph energy can provide insights into the structural properties of the graph and is often used in the design and analysis of communication networks, molecular chemistry, and social networks. Additionally, the energy of a graph is closely related to its spectrum and can be used to investigate various graph invariants, such as chromatic number, clique number, and independence number. Sinha et al. [19] introduced the splitting signed graphs as an extension of the splitting graph concept. The *splitting signed graph* of a signed graph Σ=(V,E,σ), denoted as $\Gamma \left(\Sigma \right)=\left(V_{\Gamma },\;E_{\Gamma },\;\sigma_{\Gamma }\right)$, is obtained by creating a new vertex $v^{\prime }$ for each vertex v∈V(Σ), and connecting $v^{\prime }$ to all vertices in Σ adjacent to v such that the sign of the corresponding edges is preserved, i.e., $\sigma_{\Gamma }\left(v^{\prime }u\right)\;=\;\sigma \left(vu\right)$ for all u∈N(v). In [24] authors defined, the *canonical splitting signed graph* ξ(Σ) of a signed graph Σ is defined as a signed graph ξ(Σ)=(V(ξ),E(ξ)), with $V\left(\xi \right)=V\left(\Sigma \right)\cup V{\;}^{\prime }$, where$\;V{\;}^{\prime }$ is copy of a vertex set in V(Σ) s.t. for each vertex u∈V(Σ), take a new vertex $u^{\prime }$ and E(ξ) is defined as, join $u^{\prime }$ to all the vertices of Σ adjacent to u by negative edge if $\mu_{\sigma }\left(u\right)=\mu_{\sigma }\left(v\right)=-$, where v∈N(u) and by positive edge otherwise. Throughout we write the canonical splitting signed graph as C−splittingsignedgraph. This construction is depicted in Fig. 1, Algorithm 1, Algorithm 2. A signed graph Σ is called a splitting signed graph if it is isomorphic to the splitting signed graph Γ(U)(ξ(U)) of some signed graph U, where U is referred to as the splitting root signed graph of Σ.

In this research paper, we give an algorithm to generate ξ(Σ) and its variant, the splitting root signed graph, from a given signed graph. We also perform spectral analysis on the adjacency and Laplacian matrices of the -splitting signed graph to investigate its eigenvalues and eigenvectors. Furthermore, we establish a relationship between the energy of the original signed graph and the energy of the -splitting signed graph. Our study sheds light on the properties of the -splitting signed graph, and its potential ap- plications in various domains.

The Kronecker product (or tensor product) of matrices U and W is a matrix defined as follows:

$$U⊗W=\left[\begin{array}{ccc} a_{11}W & \cdots & a_{1n}W\\ \vdots & \ddots & \vdots \\ a_{m1}W & \cdots & a_{mn}W \end{array}\right]$$

where, $U\;\in \;R^{m\times n}$ and $W\in \;R^{p\times q}$.

Theorem 1 **[25]**

*Let*U*and*W*are two square matrices such that*$U\;\in \;M^{m}$*and*$W\;\in \;M^{n}$*. If*$\mu_{i}$*is an eigenvalue of*U*with its corresponding eigenvector*$y_{i}$*, and*$\lambda_{j}$*is an eigenvalue of*W*with its corresponding eigenvector*$x_{i}$*, then*$\mu_{i}\lambda_{j}$*is an eigenvalue of the Kronecker product*U⊗W*, with the corresponding eigenvector*$y_{i}\;⊗\;x_{j}$.

The C−splittingsignedgraph concept, the algorithm for its generation, its spectral analysis and energy based comparison become an important area to be explored. These contributions can advance the field of signed graph theory and graph analysis.

In this paper, we begin by presenting a numerical interpretation for obtaining a C−splittingsignedgraph, followed by an algorithm along with its complexity analysis. Subsequently, we provide structural characterizations for identifying the splitting root signed graph, along with an accompanying algorithm and its computational complexity. Next, we establish a relationship between the spectrum of a signed graph and its C−splittingsignedgraph. Finally, we investigate the energy relation.

## Numerical interpretation to obtain C-splitting signed graph

The procedure for generating a splitting graph can be described as follows: Given a graph with n vertices, the first step is to encode an n×n symmetric adjacency matrix for the graph. Since a new vertex is created for each vertex in the original graph, the splitting graph will have a total of 2n vertices. The non-zero entries in the first row of the adjacency matrix indicate the vertices which are adjacent to the first vertex i.e. $v_{1}$. These entries in first row are also considered adjacent to vertex $v_{n+1}$ and are updated in the output matrix.

This process is repeated for each row until all rows have been processed. As a result, a 2n×2n output matrix is generated. The adjacency matrices of the original signed graph Σ and its C-splitting signed graph ξ(Σ) can be represented as follows:

$$A\left(\Sigma \right)=\left[\begin{array}{ccccc} 0 & -1 & 0 & 0 & 0\\ -1 & 0 & 1 & 0 & 1\\ 0 & 1 & 0 & 1 & 0\\ 0 & 0 & 1 & 0 & -1\\ 0 & 1 & 0 & -1 & 0 \end{array}\right]$$

and

$$A\left(\xi \left(\Sigma \right)\right)=\left[\begin{array}{cccccccccc} 0 & -1 & 0 & 0 & 0 & 0 & -1 & 0 & 0 & 0\\ -1 & 0 & 0 & 1 & 1 & -1 & 0 & 1 & 0 & -1\\ 0 & 1 & 1 & 0 & 0 & 0 & 1 & 0 & 1 & 0\\ 0 & 0 & 0 & -1 & -1 & 0 & 0 & 1 & 0 & -1\\ 0 & 1 & -1 & 0 & 0 & 0 & -1 & 0 & -1 & 0\\ 0 & -1 & 0 & 0 & 0 & 0 & 0 & 0 & 0 & 0\\ -1 & 0 & 0 & -1 & -1 & 0 & 0 & 0 & 0 & 0\\ 0 & 1 & 1 & 0 & 0 & 0 & 0 & 0 & 0 & 0\\ 0 & 0 & 0 & -1 & -1 & 0 & 0 & 0 & 0 & 0\\ 0 & -1 & -1 & 0 & 0 & 0 & 0 & 0 & 0 & 0 \end{array}\right]$$

It has been observed that the adjacency matrix, of order 2n, of the C−splittingsignedgraph ξ(Σ) can be partitioned into four matrices each of order n,

$$A\left(\xi \left(\Sigma \right)\right)=\left[\begin{array}{cc} A_{1} & B_{1}\\ B_{1} & 0 \end{array}\right]$$

where the initial matrix $A_{1}=\;A\left(\Sigma \right)$, the remaining two non zero matrices are equal, say $B_{1}$, and the fourth is null matrix. Surprisingly,

$$b_{i,j}=\left\{ \begin{array}{cc} -|a_{i,j}| & if\prod_{\begin{array}{c} i=1\\ i\;\neq \;k\;\mathrm{for}\;a_{k,j\;=\;0} \end{array}}^{n}a_{i,j}=\prod_{\begin{array}{c} j=1\\ j\;\neq \;k\;\mathrm{for}\;a_{i,k\;=\;0} \end{array}}^{n}a_{i,j}=-1\\ \left|a_{i,j}\right| & otherwise \end{array}\right.$$

where $A_{1}=\;\left[a_{i,j}\right]\;$and $B_{1}=\;\left[b_{i,j}\right]$.

Additionally, it has been discovered that the original signed graph Σ is an induced subgraph of its −splittingsignedgraph ξ(Σ).

**Algorithm 1:** Algorithm to derive the C−splittingsignedgraphξ(Σ) of a signed graph Σ.

## Computational complexity

Computational complexity analysis is an essential aspect of evaluating the performance of an algorithm. In this regard, we analyze the complexity involved in Steps 3and4 of our algorithm. In these steps, we traverse each vertex of the signed graph and examine its adjacency with all other vertices. As a result, the complexity involved in these steps is $O(n^{2})$. Again, we analyze the complexity involved in Steps 9and10 of our algorithm. In these steps, we traverse each vertex of the signed graph and examine its adjacency with all other vertices. As a result, the complexity involved in these steps is $O(n^{2})$.

Therefore, the complexity of the proposed algorithm for finding a signed split graph with a given adjacency matrix is $O\left(n^{2}\right)+O\left(n^{2}\right)=O\left(n^{2}\right)$, where n represents the number of vertices in the signed graph.

## Structural characterization to derive splitting root signed graph

Sampathkumar and Walikar [26] presented the characterization of splitting graphs, which is utilized to derive the splitting root graph.

Here, we present a structural characterization of a C−splittingsignedgraph that can be used to derive the splitting root signed graph.

Theorem 2 *Let* Σ *be a connected signed graph, then Σ is* C *-splitting signed graph if and only if the following two conditions hold:*

(a) The underlying graph $\Sigma^{u}$ is splitting graph
(b) $\sigma (vu^{\prime })\;=\;-$, whenever $\mu_{\sigma }\left(v\right)$ and $\mu_{\sigma }\left(u\right)$ both are negative in the induced graph on vertex set $V_{1}$ of Σ.

## Proof. Necessity

Let Σ be a connected C-splitting signed graph of a signed graph $\Sigma_{1}$, then it can be established that the above conditions hold for Σ. This is because $\Sigma =\xi (\Sigma_{1})$, so $\Sigma^{u}=\xi \left(\Sigma_{1}^{u}\right)$, which satisfies condition (a).

Now, during the construction of Σ from $\Sigma_{1}$, a new vertex $v^{\prime }$ is added for each vertex v in $\Sigma_{1}$. These new vertices $v^{\prime }$ are unique to each vertex v and are stored in $V_{2}$. Meanwhile, the original vertices from $\Sigma_{1}$ are stored in $V_{1}$. Based on the definition of $\xi (\Sigma_{1})$, it follows that for each vertex u that is adjacent to v and is contained in $V_{1}$, $\sigma (v^{\prime }u)=-$ whenever $\mu_{\sigma }\left(v\right)=\mu_{\sigma }\left(u\right)=-$, u∈N(v), and $\sigma (v^{\prime }u)=+$ otherwise, satisfying condition (b).

**Sufficiency:** If conditions (a) and (b) are satisfied for a signed graph Σ, then we can create a sub signed graph $\Sigma_{1}$ of Σ by including only the vertices of $V_{1}$. If we find the C-splitting signed graph of

$\Sigma_{1}$, then we find that Σ is equivalent to $\xi (\Sigma_{1})$, which leads to the conclusion of the theorem.

Theorem 3 ***[*24*]***

*For any signed graph* Σ *,* ξ(Σ)≅Γ(Σ) *if and only if the signed graph* Σ *satisfies any one of the following condition:*

(a) σ(uv)=+, whenever u and v are adjacent;
(b) σ(uv)=−, whenever u and v are adjacent and d(v)= odd for every v;
(c) Σ is heterogeneous in which both the incident vertices of each negative(positive) edges are (are not) negative.

## Deriving splitting root signed graph through structural characterization

The process for deriving the splitting root signed graph involves several steps. Firstly, it is necessary for the number of vertices, denoted as “n”, to be even in order for a splitting root signed graph to exist. If “n” is odd, then it is impossible to construct a splitting root signed graph. Once it has been established that “n” is even, an n dimensional matrix is generated to represent the given signed graph. The matrix is examined to count the number of negative and positive edges, and it is determined whether both counts are divisible by 3. If this condition is met, a splitting root signed graph can be created; otherwise, it cannot.

The next step involves calculating the number of negative and positive edges for every vertex by counting the number of −1s and1s in each row. If it is possible to partition the vertex set V(Σ) into two sets, such that the number of negative and positive edges in one set is exactly double of the other set, then a splitting root signed graph can be constructed.

If a splitting root signed graph exists, it's adjacency matrix of order n can be partitioned into four matrices of equal order$\;\frac{n}{2}$ . Let $\left[a_{i,j}\right]$, $\left[b_{i,j}\right]$, $\left[c_{i,j}\right]$ and $\left[d_{i,j}\right]$ are the four matrices of order$\;\frac{n}{2}$, then $a_{i,j}=b_{i,j}=c_{i,j}$ for $0\leq i,\;j\leq \frac{n}{2}$ and $d_{i,j}=\;0$ for $0\leq i,\;j\leq \frac{n}{2}$ i.e. $\left[d_{i,j}\right]$ is null matrix. To construct the matrix same and null, it is necessary to interchange rows and columns. This will result in a matrix of size $\frac{n}{2}\times \frac{n}{2}$ *.*

An example of the computation of a splitting root signed graph from a given signed graph will be provided in the following section.

$$A\left(\Sigma_{1}\right)=\left[\begin{array}{cccccccc} 0 & 1 & 0 & -1 & -1 & 0 & 1 & 0\\ 1 & 0 & 1 & 0 & 0 & 0 & 0 & 0\\ 0 & 1 & 0 & 1 & 1 & 0 & 1 & 0\\ -1 & 0 & 1 & 0 & 0 & 0 & 0 & 0\\ -1 & 0 & 1 & 0 & 0 & 1 & 0 & -1\\ 0 & 0 & 0 & 0 & 1 & 0 & 1 & 0\\ 1 & 0 & 1 & 0 & 0 & 1 & 0 & 1\\ 0 & 0 & 0 & 0 & -1 & 0 & 1 & 0 \end{array}\right]$$

The adjacency matrix of this signed graph, $A(\Sigma_{1})$ is a 8×8 matrix. Here the number of vertices in a signed graph is even, so we can find its splitting root signed graph. However, if the vertices are odd in numbers, then splitting root signed graph of the given signed graph will not exist. In this case, there are 6occurrences of the value −1 and 18 occurrences of the value 1 in $A(\Sigma_{1})$. Clearly, here both the values are multiple of 3, so the splitting root signed graph can be computed.

The next step is to count the number of 1s and −1s in each row of the matrix:

**Table:** 

Vertex $v_{i}$	no. of 1s	no. of −1s
1	2	2
2	2	0
3	4	0
4	1	1
5	2	2
6	2	0
7	4	0
8	1	1

With this knowledge, the vertex set is splitted into two sets, $V_{1}$ and $V_{2}$, where the number of 1s and −1sin $V_{1}\;$is double that of in $V_{2}$. In this example, $V_{1}\;$is {1,3,5,7} and $V_{2}$ is {2,4,6,8}. If the partition is successful, the splitting root signed graph exists, otherwise it does not.

Next, the splitting root graph is computed. The adjacency matrixA(ξ(Σ)), of order 2n, of the C−splittingsignedgraph can be partitioned into four equal matrices of the order n. Let $\left[a_{i,j}\right]$, $\left[b_{i,j}\right]$, $\left[c_{i,j}\right]$ and $\left[d_{i,j}\right]$ are the four matrices of order n, then $a_{i,j}=b_{i,j}=c_{i,j}$ for 0≤i,j≤n and $d_{i,j}=\;0$ for 0≤i,j≤n i.e. $\left[d_{i,j}\right]$ is null matrix.

In this example, the 8×8 input matrix is divided into four equal matrices of size 4×4. The fourth matrix constructed as null matrix and the first, second, and third matrices are made identical through transformations in rows and columns. The output matrix is 4×4.

After applying transformations such that $R_{2}\Leftrightarrow R_{7}$ and $C_{2}\Leftrightarrow C_{7}$, we get:

**Table:** 

Vertex $v_{i}$	1	7	3	4	5	6	2	8
1	0	1	0	0	−1	1	0	−1
7	1	0	1	0	0	1	0	−1
3	0	1	0	0	1	1	0	1
4	0	1	0	0	−1	0	0	0
5	−1	0	1	−1	0	0	1	0
6	1	0	1	0	0	0	0	0
2	0	1	0	0	1	0	0	0
8	−1	0	1	0	0	0	0	0

and then $R_{4}\Leftrightarrow R_{5}\;$and $C_{4}\Leftrightarrow C_{5}$, we get:

**Table:** 

Vertex $v_{i}$	1	7	3	5	4	6	2	8
1	0	1	0	−1	0	1	0	−1
7	1	0	1	0	1	0	1	0
3	0	1	0	1	0	1	0	1
5	−1	0	1	0	−1	0	1	0
4	0	1	0	−1	0	0	0	0
6	1	0	1	0	0	0	0	0
2	0	1	0	1	0	0	0	0
8	−1	0	1	0	0	0	0	0

The first, second and third matrices can be seen to be the same, and the fourth matrix is zero, which satisfies the requirements for a splitting root signed graph. The final output matrix is the splitting root signed graph:

**Table:** 

Vertex $v_{i}$	1	7	3	5
1	0	1	0	−1
7	1	0	1	0
3	0	1	0	1
5	−1	0	1	0

**Fig. 2:**
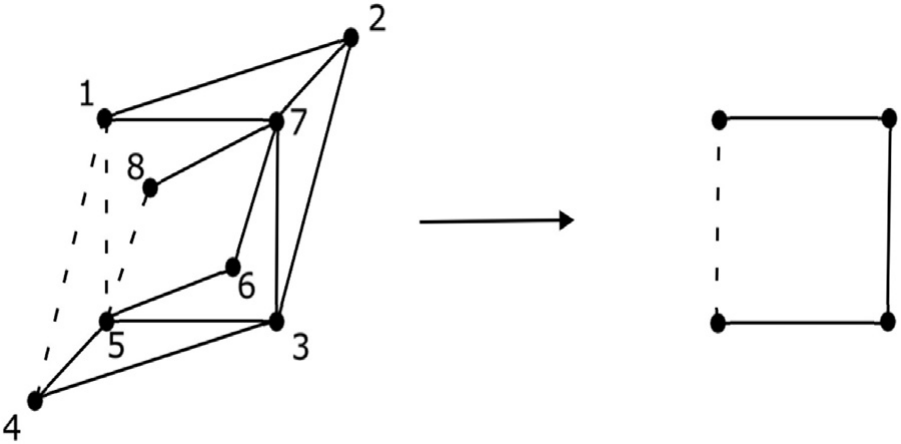
Signed graph *S*_1_ and *S* is its splitting root signed graph.

The signed graph $S_{1}$ and its corresponding splitting root signed graph S are depicted in the Fig. 2.

**Algorithm 2:** Algorithm to derive the splitting root signed graph of a given signed graph.

## Computational complexity

The algorithm calculates the total number of positive and negative edges and traverses every entry of the matrix. As a result, the complexity to count the edges is $O(n^{2})$.

In Steps 30 to 52, the complexity is $O(n^{2})$.

If the function is denoted by fun then the complexity required for the column and row transformations from Step 67 to Step 77 to construct all three matrices equal is $O(n^{3})$. If the fourth submatrix is already zero in the steps 78to91 then we use row operation to construct all other sub matrices equal. We traverse each vertex of the signed graph and examine every entry if it is equal. As a result, the complexity involved in these steps is $O\left(n^{2}\times n\times n\right)\;=\;O\left(n^{4}\right)$. Therefore, the complexity of the proposed algorithm for finding a root signed split graph with a given adjacency matrix is $O\left(n^{2}\right)\;+\;O\left(n^{3}\right)\;+\;O\left(n^{4}\right)\;+\;O\left(n^{2}\right)\;=\;O\left(n^{4}\right)$, where n represents the number of vertices in the signed graph.

## Characterization of balanced C-splitting signed graph

From Eq. (2), we can see that spectral radius of Σ will always be greater than equal to the index, i.e.

$$\rho \left(\Sigma \right)\;\geq \;\lambda_{1}\left(\Sigma \right)$$

In [4] Acharya provided the spectral criterion for balance in Σ as,

Theorem 4 *A signed graph* Σ *is balanced if and only if it is cospectral to it's underlying graph.*

It provides that the balanced signed graphs have the spectral radius equal to the index. So, here in this section we characterize the balanced C−splittingsignedgraphs.

Sampathkumar provided an important characterization of balanced signed graphs based on marking:

Theorem 5 *The balance of a signed graph* $\Sigma =(\Sigma^{u},\sigma )\;$ *can be determined if and only if there is a marking* μ *of its vertices such that the sign of each edge* vu *in* Σ *satisfies the condition* σ(vu)=μ(v)μ(u)

The operation of changing the sign of every edge in a signed graph Σ to its opposite, based on the marking μof its vertices, is called *switching* Σ with respect to μ. This operation is performed whenever the end vertices of an edge have opposite signs in $\Sigma_{\mu }.$ The concept of switching signed graphs is closely connected to the concept of balance, as indicated by the following theorem:

Theorem 6 *A signed graph* $\Sigma =(\Sigma^{u},\sigma )\;$ *is considered balanced if and only if it is equivalent under switching to its underlying graph* $\Sigma^{u}$ *.*

Theorem 7 *The* C−splittingsignedgraphs ξ(Σ) *is balanced if and only if the signed graph* Σ *is balanced.*

**Proof. Necessity:** Let us consider ξ(Σ) is balanced. As we know that Σ is a subsigned graph of ξ(Σ). This gives the result Σ is balanced.

**Sufficiency:** Assuming Σ is balanced, it follows from Theorem 5 that there is a marking μ of its vertices such that for each edge vu in Σ, σ(vu)=μ(v)μ(v). Additionally, by Theorem 6 Σ can be transformed into an all positive signed graph $\Sigma_{\mu }$. We can then extend μ to the vertex set $V_{\xi (\Sigma )}$ by setting $\mu \left(v^{\prime }\right)\;=\;\mu \left(v\right)$ and switch $\xi \left(\Sigma \right)\;to\;\xi {\left(\Sigma \right)}_{\mu^{\prime }}$*,* resulting in $\xi {\left(\Sigma \right)}_{\mu }\;=\;\xi \left(\Sigma \mu \right)$.

Since $\xi (\Sigma_{\mu^{\prime }})$ is all-positive, it is balanced. Hence, $\xi \left(\Sigma \right)\;=\;\xi {\left(\Sigma_{\mu }\right)}_{\mu^{\prime }}$ is also balanced. This establishes the sufficiency.

Remark 1 *The* C−splittingsignedgraph ξ(Σ) *is cospectral to it's underlying graph* $\xi (\Sigma^{u})$ *if and only if* Σ *is balanced.*

## Spectrum of C - splitting signed graph

In this section, we aim to find out the spectrum of the Adjacency matrix and Laplacian matrix of a C−splittingsignedgraph ξ(Σ) when (Σ)≅ Γ(Σ).

Let A(Σ) be the adjacency matrix of the signed graph Σ on n vertices, and is given as follow

$$A\left(\Sigma \right)=\left[\begin{array}{cccc} 0 & a_{12} & \cdots & a_{1n}\\ a_{21} & 0 & \cdots & a_{2n}\\ \vdots & \vdots & \ddots & \vdots \\ a_{n1} & a_{n2} & \cdots & 0 \end{array}\right]$$

Let $v_{i}^{\prime }$ be the vertex corresponding to $v_{i}$, 1≤i≤n, which is added in Σ to construct ξ(Σ), such that $N\;\left(v_{i}^{\prime }\right)\;=\;N\;\left(v_{i}\right)$, for 1≤i≤n. Then the adjacency matrix of ξ(Σ), A(ξ(Σ)), can be expressed as a block matrix with blocks as follows

$$A\left(\xi \left(\Sigma \right)\right)=\left[\begin{array}{cccccccc} 0 & a_{12} & \cdots & a_{1n} & 0 & a_{12} & \cdots & a_{1n}\\ a_{21} & 0 & \cdots & a_{2n} & a_{21} & 0 & \cdots & a_{2n}\\ \vdots & \vdots & \ddots & \vdots & \vdots & \vdots & \ddots & \vdots \\ a_{n1} & a_{n2} & \cdots & 0 & a_{n1} & a_{n2} & \cdots & 0\\ 0 & a_{12} & \cdots & a_{1n} & 0 & 0 & \cdots & 0\\ a_{21} & 0 & \cdots & a_{2n} & 0 & 0 & \cdots & 0\\ \vdots & \vdots & \ddots & \vdots & \vdots & \vdots & \ddots & \vdots \\ a_{n1} & a_{n2} & \cdots & 0 & 0 & 0 & \cdots & 0 \end{array}\right]$$

Let $\lambda_{1}\left(\Sigma \right),\;\lambda_{2}\left(\Sigma \right),\;...,\;\;\lambda_{n}\left(\Sigma \right)$ are the eigenvalues of the signed graph Σ.

We can write above adjacency matrix as,

$$A\left(\xi \left(\Sigma \right)\right)=\left[\begin{array}{cc} A\left(\Sigma \right) & A\left(\Sigma \right)\\ A\left(\Sigma \right) & 0 \end{array}\right]=\left[\begin{array}{cc} 1 & 1\\ 1 & 0 \end{array}\right]\;⊗\;A\left(\Sigma \right)$$

From here we can see that adjacency matrix A(λ(Σ)) is a Kronecker product of the matrices M and A(Σ), where $M\;=\left[\begin{array}{cc} 1 & 1\\ 1 & 0 \end{array}\right]$ . Easily we can see that $\left\{\begin{array}{cc} \frac{1+\surd 5}{2}, & \frac{1-\surd 5}{2} \end{array}\right\}$ are the eigenvalues of the matrix M.

So the adjacency spectrum of the C−splittingsignedgraph ξ(Σ) is given as $\left\{\begin{array}{cc} \frac{1+\surd 5}{2}\lambda_{i}, & \frac{1-\surd 5}{2}\lambda_{i} \end{array}\right\}$, where 1≤i≤n.

Now consider L(Σ) be the Laplacian matrix of the signed graph Σ and $\psi_{1},\;\psi_{2},\;...,\;\psi_{i}$ are the eigenvalues of the Laplacian matrix L(Σ). By some easy calculations we can find the Laplacian matrixL(ξ(Σ)) of the C−splittingsignedgraph is a Kronecker product of the matrices M andL(Σ), where$\;M\;=\left[\begin{array}{cc} 1 & 1\\ 1 & 0 \end{array}\right].$

So the Laplacian spectrum of the −splittingsignedgraph ξ(Σ) is given as $\left\{\begin{array}{cc} \frac{1+\surd 5}{2}\psi_{i}, & \frac{1-\surd 5}{2}\psi_{i} \end{array}\right\}$, where 1≤i≤n.

## **Energy of**C - splitting signed graph

In this section, we explore the connection between the energy of a signed graph Σand its C−splittingsignedgraph ξ(Σ) when ξ(Σ) *^∼^*= Γ(Σ).

Theorem 8 *Let*E(Σ)*be the energy of signed graph*Σ*and*E(ξ(Σ))*be the energy of*−splittingsignedgraphξ(Σ)*, then*E(ξ(Σ))=√5E(Σ).

**Proof.** Let $V\;=\;v_{1},\;v_{2},\;...,\;\;v_{n}$ be the vertex set of the signed graph Σ. Then adjacency matrix of Σ is given by,

$$A\left(\Sigma \right)=\left[\begin{array}{cccc} 0 & a_{12} & \cdots & a_{1n}\\ a_{21} & 0 & \cdots & a_{2n}\\ \vdots & \vdots & \ddots & \vdots \\ a_{n1} & a_{n2} & \cdots & 0 \end{array}\right]$$

Let $v_{i}^{\prime }$ be the vertex corresponding to $v_{i}$, 1≤i≤n, which is added in Σ to construct ξ(Σ), such that $N\left(v_{i}^{\prime }\right)=N\left(v_{i}\right)$, for 1≤i≤n. Then the adjacency matrix of ξ(Σ), A(ξ(Σ)), can be expressed as a block matrix with blocks as follows

$$A\left(\xi \left(\Sigma \right)\right)=\left[\begin{array}{cccccccc} 0 & a_{12} & \cdots & a_{1n} & 0 & a_{12} & \cdots & a_{1n}\\ a_{21} & 0 & \cdots & a_{2n} & a_{21} & 0 & \cdots & a_{2n}\\ \vdots & \vdots & \ddots & \vdots & \vdots & \vdots & \ddots & \vdots \\ a_{n1} & a_{n2} & \cdots & 0 & a_{n1} & a_{n2} & \cdots & 0\\ 0 & a_{12} & \cdots & a_{1n} & 0 & 0 & \cdots & 0\\ a_{21} & 0 & \cdots & a_{2n} & 0 & 0 & \cdots & 0\\ \vdots & \vdots & \ddots & \vdots & \vdots & \vdots & \ddots & \vdots \\ a_{n1} & a_{n2} & \cdots & 0 & 0 & 0 & \cdots & 0 \end{array}\right]$$

We can write it as,

$$A\left(\xi \left(\Sigma \right)\right)=\left[\begin{array}{cc} A\left(\Sigma \right) & A\left(\Sigma \right)\\ A\left(\Sigma \right) & 0 \end{array}\right]=\left[\begin{array}{cc} 1 & 1\\ 1 & 0 \end{array}\right]\;⊗\;A\left(\Sigma \right)$$

Let $\lambda_{1}\left(\Sigma \right),\;\lambda_{2}\left(\Sigma \right),\;...,\;\;\lambda_{n}\left(\Sigma \right)$ are the eigenvalues of the signed graph Σ and we can observe that the eigenvalues of$\;\left[\begin{array}{cc} 1 & 1\\ 1 & 0 \end{array}\right]$ are $\left\{\begin{array}{cc} \frac{1+\surd 5}{2}, & \frac{1-\surd 5}{2} \end{array}\right\}$.

Therefore,

$$\text{spec}\left(\xi \left(\Sigma \right)\right)=\left(\begin{array}{cc} \frac{1+\surd 5}{2}\lambda_{i} & \frac{1-\surd 5}{2}\lambda_{i}\\ n & n \end{array}\right)$$

$$E\left(\xi \left(\Sigma \right)\right)=\sum_{i=1}^{n}\left|\left(\frac{1\pm \surd 5}{2}\right)\lambda_{i}\right|=\sum_{i=1}^{n}\left|\lambda_{i}\right|\left[\frac{\surd 5+1}{2}+\frac{\surd 5-1}{2}\right]=\surd 5\sum_{i=1}^{n}\left|\lambda_{i}\right|$$

Hence,

(ξ(Σ))=√5E(Σ)

## Conclusion and scope

In conclusion, this research gives an algorithm for generating ξ(Σ) and a splitting root signed graph from a given signed graph, provided it exists. Additionally, a spectral analysis of the resulting graph is conducted by studying its eigenvalues and eigenvectors through the adjacency and Laplacian matrices. The research also establishes a relationship between the energy of the original signed graph Σ and the energy of the splitting signed graph ξ(Σ). The scope of this research could be extended by exploring further applications of the proposed algorithm and studying the properties of -splitting signed graphs in more detail.

## Ethics statements

Nil.

## Data availability

No data were used to support the findings of the study.

## CRediT authorship contribution statement

**Deepa Sinha:** Conceptualization, Supervision, Writing – review & editing. **Sandeep Kumar:** Software, Methodology, Writing – original draft.

## Declaration of Competing Interest

The authors declare that they have no known competing financial interests or personal relationships that could have appeared to influence the work reported in this paper.

## Data Availability

No data was used for the research described in the article.
